# Transcriptome analysis of signaling pathways of human peritoneal mesothelial cells in response to different osmotic agents in a peritoneal dialysis solution

**DOI:** 10.1186/s12882-019-1376-0

**Published:** 2019-05-21

**Authors:** Bin Liu, Shijian Feng, Ghida Dairi, Qiunong Guan, Irina Chafeeva, Hao Wang, Richard Liggins, Gerald da Roza, Jayachandran N. Kizhakkedathu, Caigan Du

**Affiliations:** 1 Department of Urologic Sciences, University of British Columbia, Vancouver, BC Canada; 2 General Hospital of Tianjin Medical University, No.154 Anshan Road, Heping District, Tianjin, 300052 China; 3 Department of Urology, and Laboratory of Reconstructive Urology at the Institute of Urology, West China Hospital, Sichuan University, Chengdu, Sichuan China; 4 Medicine and Medical Sciences Research Center, Deanship of Scientific Research, Umm Al Qura University, Mecca, Saudi Arabia; 5 Centre for Blood Research, and Department of Pathology and Laboratory Medicine, University of British Columbia, Vancouver, BC Canada; 6 Centre for Drug Research and Development, Vancouver, BC Canada; 7 Division of Nephrology, Department of Medicine, University of British Columbia, Vancouver, BC Canada; 8 Department of Chemistry, University of British Columbia, Vancouver, BC Canada; 9 Jack Bell Research Centre, 2660 Oak Street, Vancouver, BC V6H 3Z6 Canada

**Keywords:** Osmotic agents, Hyperbranched polyglycerol, Glucose, Transcriptome, Signaling pathways, Peritoneal mesothelial cells

## Abstract

**Background:**

Glucose is a primary osmotic agent in peritoneal dialysis (PD) solutions, but its long-term use causes structural alteration of the peritoneal membrane (PM). Hyperbranched polyglycerol (HPG) is a promising alternative to glucose. This study was designed to compare the cellular responses of human peritoneal mesothelial cells (HPMCs) to these two different osmotic agents in a hypertonic solution using transcriptome analysis.

**Methods:**

Cultured HPMCs were repeatedly exposed to HPG-based or Physioneal 40 (PYS, glucose 2.27%) hypertonic solutions. Transcriptome datasets were produced using Agilent SurePrint G3 Human GE 8 × 60 microarray. Cellular signaling pathways were examined by Ingenuity Pathway Analysis (IPA). Protein expression was examined by flow cytometry analysis and Western blotting.

**Results:**

The HPG-containing solution was better tolerated compared with PYS, with less cell death and disruption of cell transcriptome. The levels of cell death in HPG- or PYS- exposed cells were positively correlated with the number of affected transcripts (HPG: 128 at day 3, 0 at day 7; PYS: 1799 at day 3, 212 at day 7). In addition to more affected “biosynthesis” and “cellular stress and death” pathways by PYS, both HPG and PYS commonly affected “sulfate biosynthesis”, “unfolded protein response”, “apoptosis signaling” and “NRF2-mediated oxidative stress response” pathways at day 3. PYS significantly up-regulated HLA-DMB and MMP12 in a time-dependent manner, and stimulated T cell adhesion to HPMCs.

**Conclusion:**

The lower cytotoxicity of hypertonic HPG solution is in agreement with its transient and minimal impact on the pathways for the “biosynthesis of cell constituents” and the “cellular stress and death”. The significant up-regulation of HLA-DMB and MMP12 by PYS may be part of its initiation of immune response in the PM.

## Background

Peritoneal dialysis (PD) is a well-established life-saving renal replacement therapy for individuals suffering from end-stage renal disease (ESRD) [1]. In PD the waste products and excess water are removed from the body by using a hypertonic solution via the peritoneal membrane (PM). At present, crystalloid glucose at concentrations of 0.55 to 4.25% is the most commonly used osmotic agent in PD solutions such as Dianeal from Baxter (Deerfield, IL, USA). However, the incidence of ultrafiltration (UF) failure among PD patients increases with time on glucose-based PD required for effective new PD [2, 3], and UF failure is one of main reasons for PD technique failure [3]. Numerous studies have demonstrated that inclusion of glucose in the conventional PD solutions largely contributes to peritoneal membrane (PM) damage and UF failure [4–8], and its high level or long-term peritoneal load is positively correlated with an increase in all-cause and cardiovascular disease mortality, low residual renal function (RRF) and dialysate to plasma ratio of creatinine in PD patients [9, 10]. Thus, it has been proposed that a non-glucose based biocompatible osmotic agent in a PD solution may preserve long-term integrity of PM structure and function, resulting in a delay in PD technique failure.

Recently we have shown that hyperbranched polyglycerol (HPG) based PD solutions at concentrations of 2.5–15% (w/v) increases the solution osmolality to 294–424 mOsm/kg and can maintain neutral solution pH (6.6–7.4) [11–13]. In a rat model of acute PD, HPG-based PD solutions produce similar or better fluid and waste removal while preserving the PM function compared to either Dianeal (2.5% glucose) or Physioneal (PYS) (2.27% glucose) [11, 12]. Very recently, we have also demonstrated that HPG is superior to glucose for long-term preservation of the PM in a rat model of chronic PD [13, 14]. All these preclinical studies may suggest that HPG is a promising alternative to glucose in PD. However, the safety profiling of this polymer has not been fully assessed. In the current study, the objective was to investigate the global biological responses (as a measure of biocompatibility) of human peritoneal mesothelial cells (HPMCs) using a whole-transcriptome analysis after repeated exposure to HPG-based PD solution as compared to glucose-based conventional PYS.

## Methods

### Hypertonic PD solutions and cells

HPG (1 kDa) was synthesized as described previously [11, 12]. A hypertonic HPG PD solution (denoted as HPG here, approximately 402 mOsmol/kg, pH 7.4) was prepared by dissolving HPG (1 kDa, 6% w/v) in a buffered electrolye solution that had the same composition as that of PYS without glucose: sodium chloride (538 mg/100 mL), sodium lactate (168 mg/100 mL), calcium chloride dehydrate (18.4 mg/100 mL), magnesium chloride hexahydrate (5.1 mg/100 mL) and sodium bicarbonate (210 mg/100 mL) [11, 13, 14]. PYS (2.27% glucose, 395 mOsmol/L, pH 7.4) was purchased from Baxter Healthcare Co. (Deerfield, IL, USA).

The immortalized HPMCs were generated from primary HPMCs by immortalizing it with origin-deficient SV40 DNA and grown and maintained in complete K1^+/+^ medium as described previously [12]. Jurkat cells, a human leukemic T cell line, were purchased from the American Type Culture Collection (Manassas, VA, USA), and grown and maintained in RPMI 1640 medium containing 10% fetal bovine serum (FBS).

### Repeated exposure of cells with hypertonic PD solutions

Immortalized HPMCs (0.25 × 10^6^ cells/well) were seeded in 24-well plates in complete K1 medium for 18 h, followed by 6 h in either PYS or HPG solution (total 24 h or a day) in a humidified 5% CO_2_ incubator at 37 °C. This treatment cycle (18-h medium/6-h hypertonic solution) was continuously repeated for six more days/times.

### Determination of HPMC growth or death rate

At the end of 6 h treatment with either PYS or HPG in each treatment cycle, both cell death or growth of cultured HPMCs was determined by lactate dehydrogenase (LDH) release using LDH assay kit (Roche Applied Science, Laval, QC, Canada) following the manufacturers’ protocol. In brief, after each time of exposure to HPG or PYS at 37 °C under a 5% CO_2_ atmosphere, supernatants were collected from the wells, followed by complete lysis of remaining cells in a 2% Triton X-100 solution. The levels of LDH in both the supernatant and the cell lysate were measured at each time point. The cell death rate was calculated: % = LDH_s_/(LDH_s_ + LDH_ce_) × 100, whereas growth or survival rate: % = LDH_ce_/LDH_0_ × 100; where LDH_s_ represented the LDH level in the supernatant and LDH_ce_ the LDH level in the cell lysate at indicated time point, and LDH_0_ indicated the total LDH level of seeded cells in untreated cell monolayer (0 h time point).

### RNA extraction

Five experimental groups were included in this study: untreated control at day 0, PYS at day 3 and 7, and HPG at day 3 and 7. Three separate cell samples were collected from each group. In brief, after 6 h of exposure to HPG or PYS, cells were detached with a Trypsin-EDTA solution and pelleted by centrifugation. Cell pellets were snap-frozen with liquid nitrogen first, and then stored in -80 °C until use. Total RNA was extracted from the cell samples by using mirVana™ isolation kit (Ambion, Austin, TX, USA), and only the RNA samples with ≥8 of RNA Integrity Number (RIN) were used for below microarray analysis.

### Microarray and ingenuity pathway analyses (IPA)

A transcriptome database of each RNA sample was created by using Agilent SurePrint G3 Human GE 8 × 60 K Microarray kit (Agilent Technologies Canada Inc., Mississauga, ON) following a standardized protocol as described previously [15]. These RNA sequencing datasets were then imported into GeneSpring GX (Agilent, Santa Clara, CA, USA), and were analyzed with 1.3 of –log_10_ *p* value (*p* = 0.05) (*t*-test) and ≥ 1.5-fold change (FC) using IPA software (Ingenuity Systems, Redwood City, CA, USA) to compare the transcript profiling between groups.

The affected gene transcript profile (both up-regulated and down-regulated) in each group was imported into the “Canonical Pathway” frame of the IPA software to determine whether or not the signaling pathways were significantly regulated by each treatment (HPG or PYS at day 3 or 7) as compared to controls, such as at day 3 or 7 compared to at day 0, or at day 7 compared to day 3.

### Flow cytometry analysis

The cell surface expression of HLA-DMB on HPMCs was determined by using flow cytometric analysis. In brief, cells were incubated with primary rabbit monoclonal anti-HLA-DMB antibody (ab133640, Abcam, Toronto, ON, Canada), followed by labeling with secondary fluorescein isothiocyanate (FITC)-conjugated goat anti-rabbit IgG antibody (ab97050, Abcam), or with the secondary antibody only as a FITC background stain control. Stained cells were measured using a XL flow cytometer BD FACSCanto II (BD Biosciences, Mississauga, ON, Canada). The proportion of HLA-DMB positivity in the sample was presented by means of FITC light intensity (MFI) with anti-human HLA-DMB antibody.

### Western blotting analysis

Cellular levels of HLA-DMB protein in HPMCs were examined by Western blot analysis. Proteins in cellular protein extracts (50–100 μg per sample) were separated using 10% SDS-PAGE, and were then transferred onto nitrocellulose membrane. HLA-DMB protein (26–28 kDa) were identified by rabbit monoclonal anti-HLA-DMB antibody (ab133640, Abcam), and visualized by using an enhanced chemiluminescence assay (ECL: Amersham Pharmacia Biotech, Buckinghamshire, UK). Blots were re-probed using anti-Glyceraldehyde 3-phosphate dehydrogenase (GAPDH) antibody (Epitope Biotech., Vancouver, BC, Canada) to confirm the amount of loaded protein in each sample. The expression levels of the HLA-DMB were semiquantitatively determined using a densitometry, and were presented as a ratio of the HLA-DMB protein to GAPDH on the same blots.

### T cell adhesion assay

Cell adhesion of Jurket cells to monolayers of HPMCs was examined by using microscopic analysis. In brief, after repeated exposure of HPMCs with HPG, PYS or medium only (untreated control) for seven times as described above, cells (0.25 × 10^6^ cells/well) were incubated in 24-well plates overnight, followed by incubation with Jurkat cells (0.25 × 10^6^ cells/well) at 37 °C, 5% CO_2_ for 48 h. Non-adherent Jurkate cells were removed by washing four times with the culture medium. The bound Jurkat cells in each sample were counted in 10 randomly selected high power fields (hpf, 400× magnification) using a light microscope, and were averaged.

### Statistical analysis

The difference between samples was analyzed by using two-tailed t-test or analysis of variance (ANOVA) (GraphPad Prism Software, GraphPad, San Diego, CA, USA) as appropriate. Data were presented as mean ± standard derivation (SD). A *p* value ≤0.05 was considered statistically significant.

## Results

### Cell growth recovery of HPMCs was improved in treatment with HPG but not with PYS

**Fig. 1:**
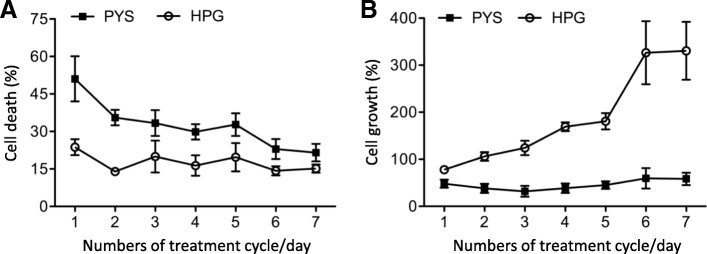
Cell death or growth of HPMCs in response to repeating exposure to hypertonic PD solutions. Immortalized HPMCs (0.25 × 10^6^ cells/well) were seeded in 24-well plates in a culture medium for 18 h, followed by 6 h in either PYS or HPG solution (total 24 h or a day) in a humidified 5% CO2 incubator at 37 °C. The level of LDH release was determined at the end of each treatment cycle (18-h medium/6-h hypertonic solution) to calculate the percentage of cell death or growth of cultured HPMCs based on the baseline in untreated control at day 0. **a** Percentage of cell death. PYS vs HPG: *p* < 0.0001, two-way ANOVA, and cell death decline over time in PYS (*p* < 0.0001, one-way ANOVA) or in HPG (*p* = 0.0161, one-way ANOVA). **b** Percentage of cell growth. HPG vs. PYS (*p* < 0.0001, two-way ANOVA), and cell growth recovery over time in PYS (*p* = 0.0382, one-way ANOVA) or in HPG (*p* < 0.0001, one-way ANOVA). Data were presented as mean ± standard derivation (SD) of four separate experiments (n = 4)

The impact of the hypertonic HPG solution compared to PYS control on HPMC survival was first determined by measuring LDH release. As shown in Fig. 1a, the death rate in HPG-treated cells was significantly lower than that in PYS control. In HPG group, the death rate was from 23.71 ± 3.18% at the first treatment to 15.16 ± 1.63% for the last treatment, while in PYS group, it was from 51.06 ± 9.05% at the first treatment to 21.55 ± 3.49% for the last treatment (*p* < 0.0001, HPG vs. PYS, two-way ANOVA, *n* = 4). The death rate in both HPG- and PYS-treated cells gradually declined following each treatment, indicated by a statistically significant difference between time points (HPG: *p* = 0.0161, PYS: *p* < 0.0001, one-way ANOVA) (Fig. 1a). When the cell growth was calculated after each treatment (Fig. 1b), data showed that there was a significant increase in cell growth in HPG-treated cells (from 77.67 ± 5.99% at the first treatment to 330.66 ± 61.60% at the last treatment, *p* < 0.0001, one-way ANOVA), whereas there was no significant change in PYS-treated cells. All these data suggested that following multiple exposures to HPG compared to PYS, there was less HPMC death in HPG groups compared to PYS group. At the same time, in each group cells became more tolerant to these non-physiologic solutions over time with the treatment.

### Less gene transcripts were affected in HPMCs by exposure to HPG than those by PYS

**Fig. 2:**
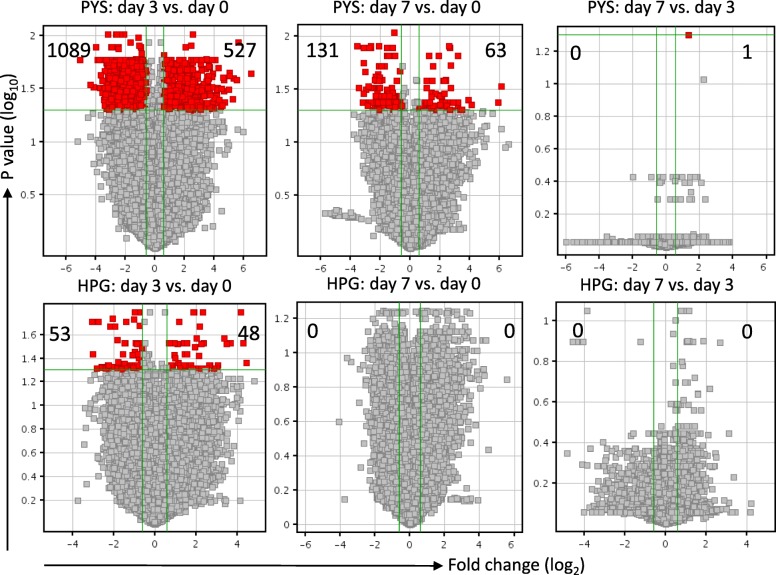
The affected transcripts in HPMCs by exposure to PYS or to HPG at day 3 and day 7. Volcano plots of microarray data (three separate samples in each group, *n* = 3) of both PYS- and HPG-treated HPMCs at day 3 and day 7 compared to untreated control, or the data at day 7 compared to those at day 3 using the Agilent Gene Spring software. Only the transcripts in these treated cells that were significantly changed (*p* ≤ 0.05; FC ≥ 1.5) compared to appropriate controls were included and presented in this analysis

In order to see the molecular responses of cultured HPMCs to HPG or PYS in a time-dependent manner, the transcriptome in the HPMCs at day 0 (as a baseline or untreated control), day 3 and day 7 in response to both HPG and PYS were analyzed by using SurePrint G3 Human GE 8 × 60 K microarray that covered total 60,000 transcripts. In PYS group as compared to the untreated control (day 0), 1089 transcripts were downregulated and 527 upregulated at day 3, and 131 downregulated and 63 upregulated at day 7 (Fig. 2, upper panel). Interestingly, the only difference in the transcriptome between at day 3 and at day 7 in PYS group was one upregulated transcript API5 (apoptosis inhibitor 5, FC = 2.4788, *p* = 0.0497). Whereas in the response to HPG, there were 53 downregulated transcripts and 48 upregulated at day 3, and there was no significant change in the transcriptome at day 7 as compared to untreated control or at day 3 (Fig. 2, bottom panel).

### Different signaling pathways were affected in HPMCs by exposure to different hypertonic PD solutions and at different time points

The canonical pathways analysis by using IPA software showed that the affected transcripts in HPMCs at day 3 of exposure to HPG solution were related to total 78 pathways, and among them 13 pathways were statistically significant (log_10_ *p* ≤ − 1.3) (Table 1). While in PYS-treated HPMCs, the transcripts were related to total 424 pathways including 6 pathways that were significant (log_10_ *p* ≤ − 1.3) at day 3, and at day 7 there were total 161 pathways including 9 pathways significant (log_10_ *p* ≤ − 1.3) (Table 1). There was little overlap in the significantly affected pathways between HPG and PYS groups or in PYS-treated cells between at day 3 and at day 7 (Table 1). It is interesting to note that the “unfolded protein response” pathway was the only significantly affected in both HPG- (log_10_ *p* = − 2.01, at day 3) and PYS-treated cells (log_10_ *p* = − 1.53, at day 7), and this pathway was less significantly affected in PYS group at day 3 (log_10_ *p* = − 0.321). Other important functional pathways, such as “CDK5 signaling”, “antigen presentation pathway”, “mitotic roles of Polo-like kinases” and “ephrin B signaling”, were either “not significantly” or “not in the list” of the affected pathways in both HPG and PYS groups at day 3, but were significantly affected in PYS-treated cells at day 7 (log_10_ *p* ≤ − 1.3) (Table 1).

**Table 1:** Significant differences in Ingenuity canonical signaling pathways between HPG and PYS

Signaling pathways	HPG (d3) (total 78)	PYS (d3) (total 424)	PYS (d7) (total 161)
Diphthamide biosynthesis	−2.09	Nil	Nil
S-adenosyl-L-methionine biosynthesis	−2.09	− 0.874	Nil
Unfolded protein response	−2.01	− 0.321	− 1.53
Role of CHK proteins in cell cycle checkpoint	−1.98	Nil	Nil
Cell cycle: G1/S checkpoint regulation	−1.86	0	Nil
Role of BRCA1 in DNA damage response	−1.7	−2.4	Nil
Cyclins and cell cycle regulation	−1.7	−0.497	Nil
Folate transformations I	−1.62	−0.456	Nil
Neuroprotective role of THOP1 in Alzheimer’s disease	−1.39	0	−0.354
Mismatched repair in eukaryotes	−1.37	−0.272	Nil
GADD45 signaling	−1.3	−0.654	Nil
Methylglyoxal degradation III	−1.3	−0.224	−1.05
DNA damage-induced 14–3-3σ signaling	−1.3	−0.224	Nil
Cell cycle: G2/M DNA damage checkpoint regulation	−0.906	−2.13	Nil
Hereditary breast cancer signaling	−1.24	−1.76	Nil
VDR/RXR activation	Nil	−1.54	Nil
Lipoate salvage and modification	Nil	−1.33	Nil
Inositol pyrophosphates biosynthesis	Nil	−1.3	Nil
CDK5 signaling	Nil	0	−1.9
Antigen presentation pathway	Nil	−0.583	−1.83
Cardiac β-adrenergic signaling	−0.325	0	−1.5
Induction of apoptosis by HIV1	Nil	0	−1.44
Mitotic roles of Polo-like kinases	−0.787	−0.711	−1.38
Dopamine-DARPP32 feedback in cAMP signaling	Nil	0	−1.33
Ephrin B signaling	Nil	−0.594	−1.3
CPCR-mediated integration of enteroendocrine signaling exemplified by an L cell	Nil	−0.351	−1.3

The total number of affected pathways was presented in each group (n = 3, three separate RNA samples). The *p* value was calculated by *t*-test as compared with the baseline control at day 0, and it was presented in a log_10_ value (− 1.30 = 0.05). Nil: not in the list of affected pathways

To further understand the differential impacts between HPG and PYS on HPMCs, the affected pathways were grouped into the “biosynthesis of cell constituents”, “cellular stress and death” and “interactions with leukocytes”. As listed in Table 2, HPG affected three “biosynthesis” pathways at day 3, including distinctively “diphthamide biosynthesis” with downregulated DPH6, and “S-adenosyl-L- methionine or cysteine biosynthesis III” with downregulated MAT2A and “dermatan, chrondroitin or heparan sulfate biosynthesis” with up-regulated CHST1. Whereas PYS affected more than twenty “biosynthesis” pathways at day 3, including “S-adenosyl-L- methionine or cysteine biosynthesis III” and the “dermatan, chrondroitin or heparan sulfate biosynthesis” as seen in HPG group but with more affected transcripts in each pathway (Table 2). At day 7, PYS only affected two pathways, which were the “dermatan, chrondroitin or heparan sulfate biosynthesis” with up-regulated CHST1 (the same as seen in HPG group at day 3) and “leukotriene biosynthesis” with downregulated MGST3 (similar to that in PYS group at day 3).

**Table 2:** Pathways for the “biosynthesis of cell constituents”

Signaling pathways	HPG (d3)	PYS (d3)	PYS (d7)
Diphthamide biosynthesis	DPH6		
Dermatan, chondroitin or heparan sulfate biosynthesis	**CHST1**	XYLT2, **CSGALNACT2**, **HS3ST1**, **DSEL**	**CHST1**
S-adenosyl-L-methionine or cysteine biosynthesis III (mammalian)	MAT2A	MAT2A, EHMT1, NSUN4	
Leukotriene biosynthesis		MGST3	MGST3
Chondroitin or dermatan biosynthesis		**CSGALNACT2**	
Glycoaminoglycan-protein linkage region biosynthesis		XYLT2	
Inositol pyrophosphate biosynthesis		**IPMK**, **IP6K2**	
D-myo-inositol (1,3,4, 5)-trisphosphate or 1D-myo-inositol hexakisphosphate biosynthesis II		**IPMK**, ITPKB, **PLCZ1**	
D-myo-inositol (1,3,4, 5, 6)-tetrakisphosphate or 3-phosphoinositide biosynthesis		**IPMK**, **PPP1R16B**, **DUSP1**, PPP1R8, **DUSP10**, **PPTC7**, DUSP12, FGFR1, RNGTT, PTPRF	
Citrulline biosynthesis		**GLS**, **ARG2**	
Lipoate biosynthesis and incorporation II		LIPT1	
Spermine or spermidine biosynthesis		**AMD1**	
Uridine-5′-phosphate biosynthesis or pyrimidine ribonucleotides de novo biosynthesis		UMPS, BLM	
Fatty acid biosynthesis initiation II		OXSM	
Serine, glycine or dTMP de novo biosynthesis		SHMT1	
Aspartate biosynthesis		**GOT1**	
CMP-N-acetylneuraminate biosynthesis I (eukaryotes)		GNE	
Ceramide biosynthesis		**KDSR**	
CDP-diacylglycerol biosynthesis 1, phosphatidylglycerol biosynthesis II (Non-plastidic) or triacylglycerol biosynthesis		TAMM41, AGPAT5	
Phosphatidylethanolamine biosynthesis II		**ETNK1**	
Dolichyl-diphosphooligosaccharide biosynthesis		ALG13	
Estrogen biosynthesis		**CYP1A1**, **HSD17B2**	
Stearate biosynthesis I (Animals)		ZADH2	

Three separate RNA samples were included in each group (n = 3). The upregulated transcripts were highlighted in bold. Others were downregulated

In the “cellular stress and death”-related pathways (Table 3), there were four pathways affected by HPG at day 3, “NRF2-mediated oxidative stress response” with downregulated DNAJC17 and EIF2AK3, “autophagy” with upregulated CTSK, “unfolded protein response” with upregulated HSPA8 and downregulated EIF2AK3, and “apoptosis signaling” with upregulated BCL2L11. In PYS-treated cells, all the pathways in the Table 3 were affected with more changed transcripts in each pathway at day 3, while at day 7, the number of the pathways was reduced to four as well as fewer changed transcripts were seen (Table 3). Overall, there were three pathways, the “NRF2-mediated oxidative stress response”, the “unfolded protein response” and the “apoptosis signaling,” commonly affected in all three groups, and “production of nitric oxide and reactive oxygen species in macrophages” was affected only by PYS at both day 3 and day 7 (Table 3).

**Table 3:** Pathways for the “cellular stress and death”

Signaling pathways	HPG (d3)	PYS (d3)	PYS (d7)
Unfolded protein response	**HSPA8**, EIF2AK3	**PPP1R15A**, **DNAJB9**, **AMFR**	HSPA8, SREBF1
Apoptosis signaling	**BCL2L11**	NAIP, BIRC6, DFFB, **BCL2L11**, PRKCA, **CASP10**	MAP3K14, **API5**
NRF2-mediated oxidative stress response	DNAJC17, EIF2AK3	FGFR1, **ABCC2**, **MAF**, **JUNB**, **MAFK**, **DNAJB9**, **MAFF**, **FOS**, DNAJC14, **GSTO2**, FKBP5, MGST3, CBR1, PRKCA	MGST3
Autophagy	**CTSK**	WDFY3, **ATG4D**, **MAP1LC3B**, ATG16L1	
Production of nitric oxide and reactive oxygen species in macrophages		**FOS**, **ALB**, **RND3**, ORM1, **PPP1R10**, FGFR, **ARG2**, PRKCA	MAP3K14, **PPP1R10**, **PPP1R3C**
Oxidative phosphorylation		**MT-ND1**, COX11, **COX8C**, UQCRFS1, SDHC	
Superoxide radical degradation		**CYGB**	
Nitric oxide signaling in the cardiovascular system		**FLT1**, FGFR1, **ARG2**, PRKCA	
Myc mediated apoptosis signaling		**YWHAE**, FGFR1	
Mitochrondrial dysfunction		**MT-ND1**, COX11, **PSENEN**, **COX8C**, UQCRFS1, SDHC	

Three separate RNA samples were included in each group (n = 3). The upregulated transcripts were highlighted in bold. Others were downregulated

In the pathways mediating “interactions with leukocytes” (Table 4), only three transcripts were affected in HPG group, including downregulated TNFSF4 for “Th1 and Th2 pathways or T helper differentiation”, up-regulated CHST1 for “LPS/IL-1 mediated inhibition of RXR function” and downregulated IL-22RA2 for “IL-22 signaling”. While in PYS group, all the “interactions with leukocytes” pathways (except of IL-22) listed in Table 4 were affected at day 3, and these pathways numbers were reduced to nine (with fewer transcripts in each pathway) at day 7. It was interesting to note that significant upregulation of both HLA-DMB and MMP12 by PYS in a time-dependent manner, which were indicated by the FC of HLA-DMB from 1.791 at day 3 to 2.122 at day 7, and of MMP12 from 2.513 at day 3 to 4.284 at day 7.

**Table 4:** Pathways for the “interactions with leukocytes”

Signaling pathways	HPG (d3)	PYS (d3)	PYS (d7)
IL-22 signaling	IL22RA2		
Th1 and Th2 Pathways or T helper cell differentiation	TNFSF4	**NOTCH2**, **NFIL3**, IL4R, **PSENEN**, **HLA-DMA**, **HLA-A**, **IL1RL1**, FGFR1, **MAF**, **HLA-DMB**, **CD274**, MAK, **BCL6**	**HLA-DMB**
LPS/IL-1 mediated inhibition of RXR function	**CHST1**	**IL1RL1**, **ABCC2**, CPT2, **HS3ST1**, **GSTO2**, MGST3, **ABCA1**	**CHST1**, SREBF1, MGST3
IL-4, Nur77 signaling in T lymphocytes, or antigen presentation		**HLA-DMA**, **HLA-A**, **HLA-DMB**, IL4R, FGFR1	**HLA-DMB**, **TAPBP**
Leukocyte extravasation signaling		**BTK**, CLDN10, **CLDN4**, FGFR1, CD44, **MMP12**, BCAR1, CTNND1, PRKCA	**MMP12**
IL-6, IL-10, IL-17A, CD40 or TNFR1/2 signaling		COL1A1, **CSNK2A2**, **FOS**, TRAF5, FGFR1, IL4R, **IL1RL1**, **ARG2,** NAIP,	MAP3K14
IL-1 or IL-8 signaling		GNB4, **FOS**, GNAQ**, RND3**, **FLT1**, FGFR1, **HBEGF**, PRKCA	MAP3K14, GNAS
GM-CSF, IL-2, IL-3, IL-7, IGF-1 or TGF-beta signaling		**RUNX1**, **CSNK2A2**, **FOS**, FGFR1, PRKCA, **CDKN1B**, **YWHAE**, **BCL6, BMP4**, SERPINE1	
IL-12 signaling or production in macrophages		**FOS**, **ALB**, ORM1, FGFR1, **MAF**, PRKCA	
Natural killer cell signaling		FGFR1, SH3BP2, KIR3DL3, PRKCA	
Cytotoxic T lymphocyte-mediated apoptosis of target cells, or granzyme B signaling		**HLA-A**, DFFB	
Antiproliferative role of TOB in T cell signaling		**TOB1**, **CDKN1B**	

Three separate RNA samples were included in each group (n = 3). The upregulated transcripts were highlighted in bold. Others were downregulated

### Verification of HLA-DMB expression in HPMCs after exposure to hypertonic PD solutions

**Fig. 3:**
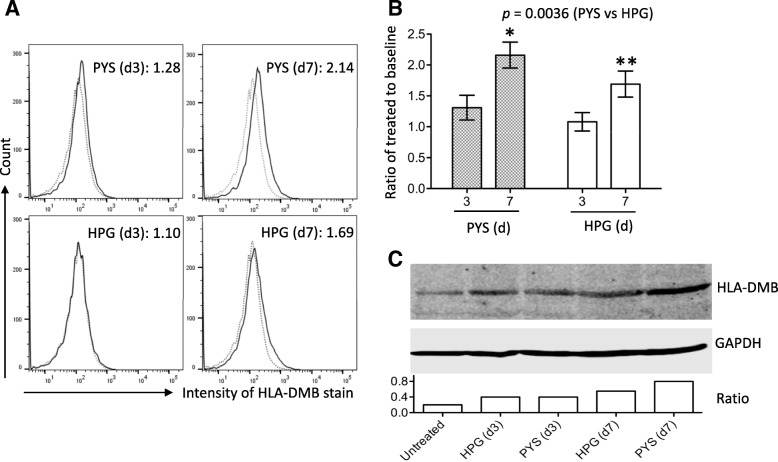
Upregulation of HLA-DMB expression on HPMCs after exposure to PYS or to HPG at day 3 and day 7. The mean of fluorescence intensity (MFI) of HLA-DMB staining on HPMCs after repeating exposure to PYS or to HPG at day 3 and day 7 compared to untreated control at day 0 (as a baseline) was measured by using flow cytometric analysis. The ratio of MFI on treated cells to its baseline was presented as fold change of HLA-DMB expression. **a** Data were presented as a typical histogram of HLA-DMB stain (solid line) in each group compared to the baseline (MFI: 126, dotted line), showing the fold change of HLA-DMB expression. **b** Data were presented as mean ± SD of four separate experiments (*n* = 4). *p* = 0.0036 (PYS vs. HPG, two-way ANOVA). *p = 0.0011 (PYS: day 3 vs. day 7, t-test). **p = 0.0032 (HPG: day 3 vs. day 7, t-test). (C) The total cellular levels of HLA-DMB staining on HPMCs after repeating exposure to PYS or to HPG at day 3 and day 7 compared to untreated control at day 0 were determined by Western blot analysis. Equal amount of protein (100 to 150 μg) extracted from whole cell pellets was fractioned by 10% of SDS-PAGE, and HLA-DMB protein bands were identified based on specifically binding of anti-HLA-DMB antibody, and their molecular size (26–28 kDa) (upper panel). The protein content in each sample was confirmed by re-probing the blot with anti-GAPDH antibody (middle panel) and was measured by densitometry. Imaging data are a representative of three separate experiments. The ratio of HLA-DMB band to GAPDH band from the same sample on the same blot was presented (bottom panel)

HLA-DMB is HLA class II beta chain that is required for MHC class II/peptide complex formation in the antigen presentation to T cells [16]. As mentioned above, the expression of this molecule in HPMCs was significantly up-regulated by the exposure to PYS. There was also a 1.57-fold increase in HLA-DMB expression in HPG-treated cells at day 3 and a 2.43-fold increase at day 7 but these changes were not statistically different as compared to the baseline control in this sample size (*n* = 3). These microarray data were further confirmed using both flow cytometric and Western blot analyses. The increased cell surface levels of HLA-DMB were significantly higher in PYS-treated HPMCs than those in HPG-treated cells (*p* = 0.0036, two-way ANOVA, *n* = 4), evidenced by the ratio of PYS-treated cells to the baseline from 1.31 ± 0.2 at day 3 to 2.16 ± 0.21 at day 7, whereas in HPG group it was 1.08 ± 0.16 at day 3 to 1.69 ± 0.21 at day 7 (Fig. 3b). In Western blot analysis of total cellular HLA-DMB protein levels (Fig. 3c), HLA-DMB levels in PYS-treated cells were higher than those in HPG-treated cells at both time points. Taken together, these data suggested that the hypertonic PYS solution induced higher expression of HLA-DMB than HPG, which was significantly associated with “antigen presenting pathway” in PYS group at day 7 (Table 1).

### Increased T cell adhesion to HPMCs after repeated exposure to hypertonic PD solutions

**Fig. 4:**
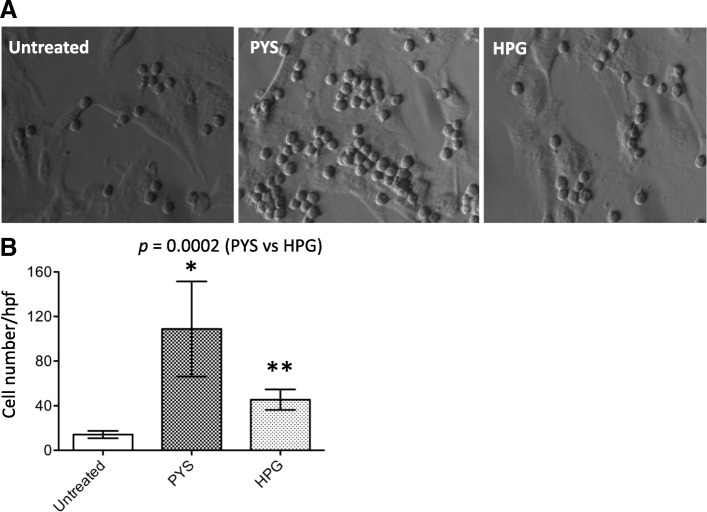
T cell adhesion to HPMCs after exposure to PYS or to HPG for 7 days. A monolayer of HPMCs after treatment with PYS or HPG for 7 days was co-cultured with an equal number of Jurkat T cells for 48 h. **a** Imaging data were presented as a typical microscopic view of adhesive T cells, indicated by small size and spherical in shape in each group. **b** Data were presented as mean ± SD of ten separate experiments (*n* = 10). *p* = 0.0002 (PYS vs. HPG, t-test). *p < 0.0011 (PYS vs. Untreated, t-test). **p < 0.0011 (HPG vs. Untreated, t-test)

To investigate the functional implications of increased HLA-DMB and perhaps others such as MMP12 in HPMCs after exposure to hypertonic solutions, the level of T cell adhesion to these cells was examined. After repeated exposure to HPG or PYS for 7 days as described previously, HPMCs were co-cultured with Jurkat cells in a 1:1 ratio for 48 h. After removal of all the non-adherent cells, the number of Jurkat cell sticking to HPMCs was counted. As shown in Fig. 4, the number of Jurkat cells in PYS was 108.8 ± 42.78 per hpf that was significantly higher than 45.5 ± 9.23 per hpf in HPG group (*p* = 0.0002, two-tailed t-test, *n* = 10), whereas in untreated cells with medium only it was 14.2 ± 3.39 per hpf. These findings indicated the positive correlation of up-regulated expression of HLA-DMB and the others with an increase in T cell adhesion to the HPMCs after exposure to hypertonic solutions, and the effect of PYS was higher than HPG in this in vitro system.

## Discussion

Long-term exposure to a glucose-based PD solution is associated with inflammation and fibrosis in the PM [17], which consequently result in its gradual functional impairment characterized by progressive reduction in solute transport or UF failure [18]. However, the cytotoxic mechanism of the peritoneal response to a hypertonic PD solution has not yet been fully elucidated. A previous study has demonstrated that TGF-β1/Smad signaling pathway is activated by the hypertonic solution in a chronic PD rat model, and possibly participates in the pathogenesis of PD related peritoneal fibrosis [19]. Recent findings have suggested that epithelial-mesenchymal transition (EMT) of peritoneal mesothelial cells may play an important role in the failure of PM function [20]. However, it is still not known that how these possible signaling pathways contribute to the inflammation and fibrosis seen in the peritoneum of PD patients. Thus, in this study, our primary goal was to reveal the biological pathways affected by a hypertonic solution in cultured HPMCs by transcriptome analysis. Since our previous studies demonstrated that the compact HPG is a superior biocompatible osmotic agent compared to glucose in rat models of PD [11–14], evidenced by the fact that the HPG-based hypertonic solution induces less PM damage than glucose-based PD solutions. However, the pathways mediating the difference in the “toxicity” between the HPG- and the glucose-based hypertonic PD solutions in the peritoneum have not been fully investigated. Thus our second goal in this study was to compare the pathways affected by the HPG solution with those by PYS in HPMCs using a whole-transcriptome analysis that is an emerging and continually growing field in assessing the safety of drugs or chemical risk assessment [21, 22]. This approach allows a more comprehensive understanding and a fuller knowledge of the “biocompatibility” of the agents/chemicals than those just based on several “biomarkers”.

Cell death is an essential attribute of tissue damage. In this study, we showed that the levels of cell death in both HPG and PYS groups at different time points were positively correlated with the numbers of changed transcripts (both up-regulated and down-regulated) in HPMCs (Figs. 1 and 2). The IPA pathway analysis did not link the cell death induced by the hypertonic solution (either HPG or PYS) to any of these significantly affected pathways (Table 1). Instead, the “dermatan, chrondroitin or heparan sulfate biosynthesis”, “unfolded protein response”, “apoptosis signaling” and “NRF2-mediated oxidative stress response” were commonly affected by HPG at day 3 and by PYS at both time points, and in addition to more pathways by PYS at day 3 with more cell death (Tables 2 and 3). Interestingly, there was no single transcript commonly found in these pathways between HPG and PYS groups (Tables 2 and 3). These data suggest that the cell death induced by either hypertonic HPG or PYS solution may be primarily mediated by the disruption of “sulfate biosynthesis” and activation of “unfolded protein response”, “apoptosis signaling” and “NRF2-mediated oxidative stress” but their actions may be different at different time points due to regulating different transcript expression.

It is interesting to note that over the time of exposure to PYS, cell death was remarkably decreased, or cells gradually became resistant to PYS-induced cell death (Fig. 1). At the same time, we found that only one transcript – API5 (apoptosis inhibitor 5) was significantly upregulated at day 7 as compared to day 3 (Fig. 2). We also noticed significant up-regulation of PPP1R10 (protein phosphatase 1 regulatory subunit 10) at both day 3 and day 7 as compared to untreated control (Table 3). According to the GeneCards® Human Gene Database (https://www.genecards.org/), PPP1R10 plays a role in cell cycle progression, DNA repair and apoptosis by negatively regulating the activity of protein phosphatase 1, a proapoptotic activator [23, 24]. Thus, it is reasonable to hypothesize that up-regulation of both PPP1R10 and API5, apoptosis inhibitors, may be part of the mechanism by which cell death resistance is induced by repeated exposure to PYS in this experimental system.

Chronic inflammation is a hallmark of structural alterations and dysfunction of the PM during PD [25], and exposure of a non-physiological, hypertonic PD solution is considered as a non-infective factor leading to chronic inflammation, macrophage infiltration and oxidative stress in the PM, resulting in UF failure in PD patients [26]. However, the pathways by which the inflammation is initiated by a hypertonic PD solution in the PM are largely unknown. The transcriptome analysis in this study revealed many inflammation-related transcripts predominantly affected in PYS at both day 3 and day 7 (Table 4). The most notable of them were up-regulation of both HLA-DMB and MMP12, which were persistently induced by PYS at both time points (Table 4). The up-regulation of HLA-DMB protein was also confirmed by both flow cytometric and Western blot analyses (Figs. 3 and 4). HLA-DMB is the beta chain of the non-classical HLA class II heterodimer (HLA-DM) that plays a central role in the peptide loading of MHC class II molecules and initiation of an immune response to the antigen stimulation [27, 28], and increases T cell adhesion to HPMCs (Fig. 4). This observation is supported by evidence in literature showing that HPMCs participate in antigen presentation, and T cell growth and adhesion [29, 30], and the antigen presentation in the peritoneum stimulates resident population of effector memory T cells in PD patients [31]. These peritoneal T cells, particularly CD8^+^ cytotoxic T cells, could play a destructive role for PM damage [32]. MMP12 is a potent elastase, and its function is involved in many inflammatory conditions, such as the inflammatory process of respiratory diseases (chronic obstructive pulmonary disease, pulmonary fibrosis and asthma) [33], and mediation of inflammation and IL-13-induced liver fibrosis [34] and inflammatory arthritis [35]. In this study, we also showed increased T cell adhesion and up-regulated HLA-DMB expression in HPMCs after exposure to PYS (Fig. 4). All this evidence may indicate that both HLA-DMB and MMP12 are the local inflammatory mediators of the PM induced by exposure to hypertonic PYS or perhaps other PD solutions.

Although positive correlations were obtained in our transcriptome analysis, the present study has several limitations. Firstly, the data were collected from cultured HPMCs, which may not represent the mesothelial cells in the PM of PD patients. The findings from this study need to be verified in clinical studies as well as in other experimental systems such as preclinical animal models. Secondly, we only tested PYS in this study. The results could be different when HPMCs are treated with other types of PD solutions such as Dianeal solutions or Extraneal (Icodextrin). Thirdly, we only measured the levels of the transcripts using microarray chips, which may not exactly reflect their protein levels. Also, the pathways due to the change of the transcripts were predicted using a bioinformatics analysis tool – IPA. Further studies are required to confirm the changes in the protein levels and their impacts on cell death, survival and inflammatory initiation.

## Conclusions

We have previously demonstrated the superior biocompatibility of HPG-based PD solutions over conventional glucose-based PD solutions in rat models of PD [11–14], but the molecular mechanisms have not been investigated. In this study, for the first time, we demonstrated that the lower cytotoxicity of the HPG solution to HPMCs in comparison to PYS correlated with its transient and mild effect on the pathways for the “biosynthesis of cell constituents” and the “cellular stress and death”. Both HPG and PYS commonly affected but in different characteristic pathways for “sulfate biosynthesis”, “unfolded protein response”, “apoptosis signaling” and “NRF2-mediated oxidative stress response”. In addition, exposure to PYS but not to HPG significantly upregulated HLA-DMB and MMP12 and increased T cell adhesion, which may be part of mechanism for the initiation of immune response and inflammation in the PM by the exposure to a glucose-based PD solution. We also have to acknowledge that less gene transcripts affected by HPG in cultured HPMCs may not necessarily reflect its superior biocompatibility over glucose in PD patients. Further evaluation of these findings in animal models of PD and in clinic is needed, which may provide a new therapeutic strategy to reduce the inflammation and structural alteration of the PM during PD.

## Abbreviations

ANOVA: Analysis of variance
EDTA: Ethylenediaminetetraacetic acid
ESRD: End-stage renal disease
FBS: Fetal bovine serum
FC: Fold change
FITC: Fluorescein isothiocyanate
GAPDH: Glyceraldehyde 3-phosphate dehydrogenase
HLA: Human leukocyte antigen
HPG: Hyperbranched polyglycerol
HPMC: Human peritoneal mesothelial cell
LDH: Lactic acid dehydrogenase
MFI: Means of FITC light intensity
MMP: Matrix metalloproteinase
PD: Peritoneal dialysis
PM: Peritoneal membrane
PYS: Physioneal
RNA: Ribonucleic acid
RRF: Residual renal function
UF: Ultrafiltration
